# The association between transformational leadership and teachers' creativity: professional resilience and job satisfaction as mediators

**DOI:** 10.3389/fpsyg.2024.1514621

**Published:** 2024-12-30

**Authors:** Ayça KAYA

**Affiliations:** Department of Education Sciences, Haliç University, Istanbul, Türkiye

**Keywords:** transformational leadership, teachers' creativity, creativity, professional resilience, resilience, job satisfaction, teacher

## Abstract

This study investigated the associations between transformational leadership, teachers' job satisfaction, professional resilience, and creativity. The sample included 417 teachers of public and private schools in Istanbul. The data collection tools were “Transformational Leadership Scale”, “Teachers' Professional Resilience Scale”, “Job Satisfaction Scale”, and “Organizational Creativity Scale”. The data analysis was conducted on SPSS and AMOS. A structural equation model (SEM) was employed to test the research hypotheses. The findings suggested that transformational leadership significantly predicted teachers' job satisfaction and professional resilience. However, transformational leadership did not have a statistically significant effect on teachers' creativity. Additionally, teachers' professional resilience was a significant predictor of creativity while job satisfaction was not. Lastly, professional resilience mediated the association between transformational leadership and creativity. Drawing on these findings, it can be concluded that the prevalence of transformational leadership in schools could foster teachers' creativity and professional resilience. Thus, principals should improve their transformational leadership skills and develop strategies to enhance teachers' creative potential. Transformational leadership plays a critical role in fostering teachers' job satisfaction and professional resilience and encourage creativity.

## Introduction

The association between transformational leadership and teachers' creativity is multifaceted, as it is significantly influenced by professional resilience and job satisfaction. Creativity in education is essential for fostering innovative teaching methods, enriching learning processes, and enhancing student outcomes (Zhang and Bartol, 2010; Hidayat and Tjahjono, 2023). Transformational leadership plays a crucial role in this process by enhancing teachers' performance and motivation through the articulation of a shared vision, values, and goals in an inspiring manner. First conceptualized by Bass (1990), transformational leadership encompasses four key components: individualized consideration, intellectual stimulation, inspirational motivation, and idealized influence. These dimensions enable leaders to inspire employees by fostering innovation and creativity within the workplace (Bass and Avolio, 1994; Leithwood and Sun, 2012). This leadership style is particularly relevant in educational settings where innovation and adaptability are critical for addressing the dynamic needs of teachers and students (Tse et al., 2018; Zhang et al., 2018). The existing literature highlights that transformational leadership not only enhances teachers' creativity but also addresses critical professional outcomes such as resilience and satisfaction. For example, Avolio and Gardner (2005) emphasize that transformational leadership cultivates a positive organizational climate, which is conducive to creativity and innovation. Furthermore, it fosters an environment where teachers feel empowered to take initiative and experiment with new teaching methods, thereby promoting professional growth and development (Shin and Zhou, 2003; Herman and Chiu, 2014). This supportive environment not only encourages individual creativity but also contributes to building a collaborative and innovative school culture where collective growth becomes possible. While transformational leaders help teachers discover and develop their individual potential, they also turn school culture into positive (Sui et al., 2012). On the other hand, teachers' creativity is of great importance to developing innovative and effective teaching methods. Teachers' creativity enables them to teach more interactively and interestingly, which in turn fosters students' academic achievement. The influence of transformational leadership on teachers' creativity is mediated by factors such as job satisfaction and professional resilience. While job satisfaction refers to the teachers' satisfaction out of teaching, professional resilience refers to the capacity to work under stressful and challenging conditions (Promchart and Potipiroon, 2020). Professional resilience, the ability to adapt and succeed in the face of challenges, is crucial for teachers to maintain high levels of creativity and innovation. By providing support, encouragement, and a clear vision, transformational leaders can increase teachers' resilience, enable them to overcome obstacles and remain creative in their teaching practices. Bogler (2001) reported that transformational leadership improved teachers' creative thinking and problem-solving skills increasing their job satisfaction and professional resilience. However, it is crucial for transformational leaders to give individualized consideration to teachers, understand their needs, and foster a supportive environment (Zhang et al., 2022). Conversely, there are also convincing evidence that transformational leadership does not have a direct effect on creativity but an indirect effect through job satisfaction (Hidayat and Tjahjono, 2023). In another study conducted on vocational high school teachers, it was reported that transformational leadership and satisfaction had direct effects on creativity and also transformational leadership enhances creativity through satisfaction (Ripki et al., 2020). Consistently, Rahmatika and Saragih (2023) revealed that transformational leadership had a significant effect on innovative work behavior with the mediator role of job satisfaction. Drawing on these findings, it can be concluded that transformational leaders foster an environment in which teachers feel satisfied and valued which in turn encourage them to engage in innovative and creative activities (Rahmatika and Saragih, 2023). Nguni et al. (2006) also suggested that transformational leadership had a significant effect on teachers' job satisfaction, organizational commitment, and organizational citizenship behaviors. The study revealed that job satisfaction mediated the association between transformational leadership and organizational outcomes and showed that satisfied teachers were more likely to exhibit creative and innovative behaviors.

The current study examines the association between transformational leadership and teachers' creativity. Additionally, investigates the mediating roles of professional resilience and job satisfaction on this relationship. Previous research indicated that job satisfaction and professional resilience significantly mediated the link between transformational leadership and teachers' creativity. Unveiling the leadership strategies effective for educational leaders to maximize teachers' creative potentials is a critical step in developing educational policies and practices (Fernandes et al., 2022) and fostering sustainable success and innovative teaching methods (Ripki et al., 2020). Transformational leaders play a crucial role in creating a supportive and satisfying work environment, which in turn encourages creativity and innovation among teachers. Understanding and leveraging these mediating factors helps educational leaders enhance teachers' creative capacities, ultimately contributing to improved educational outcomes. Teachers' creativity is critical to fostering innovative and effective teaching methods. By integrating creativity into their instructional practices, teachers enrich students' learning experiences, improve academic performance, and sustain motivation through dynamic and engaging educational practices (Hidayat and Tjahjono, 2023; Zhang et al., 2022). This underscores the importance of nurturing teacher creativity as a cornerstone for advancing both student success and overall educational quality.

### Transformational leadership

Transformational leadership is a dynamic and effective leadership style that focuses on inspiring and motivating followers to reach their full potential and exceed their own expectations. According to Bass and Avolio (1994), transformational leadership is characterized by its ability to inspire, innovate, and motivate followers toward achieving collective goals. This leadership approach fosters significant change in both individuals and organizations by promoting a shared vision, supporting innovation, and encouraging personal and professional growth. Transformational leaders are viewed as role models who inspire trust and respect, articulate a clear and compelling vision, and encourage creativity and innovation among their followers. Additionally, they provide individualized support and encouragement, tailoring their approach to meet the unique needs of each individual. Often described as charismatic and visionary, transformational leaders have the ability to align the goals of their followers with a shared future that resonates deeply with their values and aspirations (Bush, 2018; Quiros, 2020). One of the defining characteristics of transformational leadership is its emphasis on organizational behavior, culture, and the transformation of individuals. Transformational leaders are adept at creating a sense of purpose and direction that helps align followers' goals and motivations with the organization's overarching vision. This harmony is crucial to fostering a cohesive and motivated workforce committed to achieving collective goals (Bush, 2018). Additionally, transformational leaders place a high value on ethics and integrity, set accountable standards, and lead by example, thereby building the trust and credibility necessary for effective leadership (Quiros, 2020). Beyond individual followers, the influence of transformational leadership extends to group dynamics and organizational outcomes. For example, Kandemir (2024) highlights that transformational leadership fosters innovation and collaboration while reducing workplace exclusion by enhancing school effectiveness. This aligns with the broader understanding that transformational leaders positively influence organizational culture by promoting inclusivity and shared goals (Alzoraiki et al., 2024; Joo and Lim, 2013; Marks and Printy, 2003). Such leadership not only addresses organizational challenges but also creates a culture that supports collective growth and effectiveness. Research demonstrates that transformational leadership is positively associated with group cohesion, empowerment, and overall effectiveness. By empowering followers to take ownership of their tasks and fostering collaboration, transformational leaders cultivate an environment of enhanced collective efficacy, which ultimately improves group performance (Fareed et al., 2022; Jung and Sosik, 2002). These findings underscore the dual role of transformational leadership in addressing workplace challenges while simultaneously building a culture of innovation and mutual trust. Transformational leadership is associated with increased creativity and innovation at both individual and organizational levels. Leaders who adopt this style are able to create an environment that encourages creative thinking and problem solving, which in turn increases organizational innovation and competitiveness (Gumusluoglu and Ilsev, 2009). Transformational leadership also has significant effects on employee well being. There is convincing evidence that transformational leadership positively predicts aspects of wellbeing such as satisfaction, commitment, and overall psychological health. This effect is often mediated by factors such as psychological empowerment and supportive work environments fostered by transformational leaders. However, it is noteworthy that the link between transformational leadership and well being is complicated and may be influenced by various mediating and moderating factors (Arnold, 2017).

Previous literature suggests that transformational leadership lead to favorable outcomes in educational settings, as well. Transformational school leaders engage in practices that improve conditions within the school environment, enhance teacher motivation and performance, and ultimately contribute to better student outcomes. These practices include setting high expectations, providing individualized support, and fostering a collaborative school culture (Leithwood and Sun, 2012). The effectiveness of transformational leadership in educational settings underscores its versatility and applicability across different organizational contexts. Consequently, transformational leadership is a powerful and effective style capable of generating significant positive changes in individuals, groups, and organizations. Transformational leaders can inspire their followers to achieve exceptional results by promoting a shared vision, supporting innovation, and prioritizing ethical behaviors (Deinert et al., 2015; Siangchokyoo et al., 2020).

### Creativity

Teachers' creativity is critically important for the development of innovative and effective teaching methods. By fostering critical thinking and problem-solving skills, creative teachers significantly enhance students' learning experiences, which are essential for both academic and personal success. Understanding the factors that influence teachers' creativity, such as leadership styles, is therefore crucial for developing effective educational strategies (Hidayat and Tjahjono, 2023; Zhang et al., 2022). Creative teachers not only enrich students' learning processes and improve their academic outcomes but also sustain their motivation to learn. Furthermore, promoting creativity in educational settings supports teachers' personal and professional development while simultaneously improving the overall quality of education. Teachers' creative skills play a pivotal role in making educational processes dynamic and effective by continuously renewing and improving teaching methods (Hidayat and Tjahjono, 2023). To ensure innovation and continuous development in education systems, the significance of creativity should be appreciated. Employing innovative teaching methods help students develop critical thinking, problem-solving and creative thinking skills. Teachers' creativity enables students to actively participate in courses and better understand the content (Sui et al., 2012) and refers to a set of skills to create more engaging and motivating learning environments for students. It increases students' sense of curiosity and desire to learn, contributing to them becoming more active and participatory individuals (Bogler, 2001). Thus, developing teachers' creativity is the key to improving quality and effectiveness in teaching (Zhang et al., 2022). When teachers boost their creativity, they have a higher professional commitment and satisfaction which promotes their capacity to cope with stress and create more sustainable educational settings.

Creative teachers constantly innovate and improve their course materials and teaching strategies providing a great advantage in attracting students' attention and ensuring their active participation in the learning process (Ripki et al., 2020). Additionally, creative teachers can better respond to students' different learning styles and needs, making learning experiences personalized and effective (Promchart and Potipiroon, 2020). Therefore, educational institutions need to take strategic steps to maximize teachers' creative potential. Enhancing teachers' creativity requires a culture that promotes creative thinking. In such a culture, teachers are allowed to generate and implement these ideas (Hidayat and Tjahjono, 2023). Professional development programs and workshops can be an effective way for teachers to learn creative skills and innovative teaching methods (Zhang et al., 2018). These activities facilitate teachers to gain insights into new pedagogical approaches and technologies, thereby enhancing their creativity and professional competencies. The promotion of creativity is significant not only for teachers but also for students. Creative thinking skills contribute to students' professional and personal success in future (Ripki et al., 2020). Therefore, educational organizations should develop and implement strategies to maximize both teachers' and students' creativity (Thomas et al., 2020). Teachers' creativity is of a critical factor in the development of innovative and effective teaching methods. Creative teachers enrich the learning processes for students and improve their academic achievement. Thus, encouraging teachers' creativity is of great importance for the success and sustainable development of educational systems (Bogler, 2001). Adopting a creative and innovative approach within educational systems contribute significantly to the development of both teachers and students in the long term.

### The association between transformational leadership and creativity

Research on the association between transformational leadership and creativity represent a growing field particularly in educational research. Transformational leadership is a leadership style allowing leaders to increase creativity by motivating individuals with a visionary, inspiring and innovative approach. Previous research suggested that transformational leadership enhanced employee creativity and this link was mediated by psychological empowerment, innovation support and the provision of learning opportunities (Al Harbi et al., 2019; Sui et al., 2012). On the other hand, Apiani et al. (2023) found that transformational leadership had a positive influence on teacher creativity which promoted the development of innovative teaching methods. Similarly, Zhang et al. (2019) demonstrated that transformational leadership contributed to teachers' information sharing and creative thinking and this improved teachers' creative skills. Azim et al. (2019) found that transformational leadership enhanced employees' engagement in creative processes with the mediating effect of creative self-confidence. In another study, Alarifi and Alharbi (2019) demonstrated that transformational leadership increased employees' perception of psychological empowerment which in turn improved their creativity and innovative thinking capacity (Bogler, 2001). Promoting individuals' self-confidence, psychological empowerment positively contributed to creative thinking and developing innovative approaches.

In literature there is convincing empirical evidence that using intellectual stimulation, individualized consideration, and inspirational motivation, transformational leaders promotes teachers' creativity and innovative thinking (Tse et al., 2018; Ripki et al., 2020). On the other hand, Mahmood et al. (2019) found that transformational leaders promoted teachers' creativity and the effect was further strengthened by complex tasks and innovation support. Based on these, it can be concluded that when teachers are supported in solving complex problems and adopting innovative teaching strategies, they become more creative.

Transformational leadership stands out as an effective leadership model that increases creativity (Kasimoglu and Ammari, 2020). Through mechanisms such as psychological empowerment, creative self-confidence, and innovation support, transformational leaders enable teachers to unleash their creative potential and develop more innovative and effective teaching practices (Chaubey et al., 2019). Moreover, creative teachers can better respond to students' different learning styles and needs, which makes learning experiences personalized and effective (Promchart and Potipiroon, 2020). The adoption of transformational leadership strategies can pave the way for creative thinking and innovation in educational settings, thereby increasing student achievement and teacher motivation (Qian and Kee, 2023). In other words, enhancing teacher creativity is the key to improving quality and effectiveness in education (Ma et al., 2020; Zhang et al., 2022).

### Job satisfaction and professional resilience as mediators

Transformational leadership has a key role in boosting employees' performance and creativity. This leadership approach supports employees' professional resilience and job satisfaction, enabling them to be more effective in the workplace. Research shows that transformational leaders promote creativity through job satisfaction and professional resilience (Djourova et al., 2020; Kabir, 2022). Thanks to the inspiring and individualized approaches of their leaders, employees improve their ability to cope with challenges and can be more innovative (Siswanto and Yuliana, 2022; Tentama et al., 2020). Additionally, in this process, job satisfaction allows employees to focus more on their work and contribute creatively to work processes (Hidayat and Tjahjono, 2023; Fareed et al., 2022). Professional resilience is a trait that increases employees' capacity to cope with the stress and challenges in the work environment. Transformational leaders enable employees to approach the challenges at work more resiliently supporting their professional resilience (Fathizadeh et al., 2020; Hidayat and Tjahjono, 2023).

Employees with higher professional resilience maintain their job satisfaction with the support of their leaders and become more creative (Chen et al., 2021; Kabir, 2022) which implies that professional resilience functions as a mechanism that strengthens the influence of transformational leaders and increases employees' creative thinking capacity. Job satisfaction is the satisfaction individuals get out of their jobs and stands out as a mediator factor that increases the effect of transformational leadership on job performance and creativity. Employees with higher job satisfaction are more committed to their jobs and can come up with more creative solutions (Siswanto and Yuliana, 2022; Fareed et al., 2022). Additionally, higher job satisfaction enables employees to cope more easily with stress and maximizes their creativity with leadership support (Chen et al., 2021; Hidayat and Tjahjono, 2023). Transformational leaders positively influence employees' job satisfaction, allowing them to contribute more to work processes and develop creative solutions (Kabir, 2022; Tentama et al., 2020). Thus, job satisfaction contributes to the establishment of a strong link between transformational leadership and creativity. Based on these, it can be concluded that the mediating role of professional resilience and job satisfaction is one of the key factors strengthening the association between transformational leadership and creativity. While professional resilience enables employees to cope with difficulties, job satisfaction increases their professional commitment and promotes their creativity (Djourova et al., 2020; Kabir, 2022). Research also suggests that professional resilience and job satisfaction significantly boost the influence of transformational leadership on performance (Fathizadeh et al., 2020; Chen et al., 2021). Shortly, professional resilience and job satisfaction stand out as influential mechanisms that enable transformational leaders to promote creativity.

### Significance and objective of the study

This study investigates the association between transformational leadership and teachers' creativity through the mediating role of professional resilience and job satisfaction. Previous literature suggest that transformational leadership positively affects employees' creativity by motivating them, providing support, and creating an environment conducive to innovative thinking (Chen et al., 2021; Kabir, 2022; Siswanto and Yuliana, 2022). Previous research also establish that transformational leadership has a direct positive influence on teachers' professional resilience and job satisfaction, which play a critical role in enhancing individual creativity (Fathizadeh et al., 2020; Hidayat and Tjahjono, 2023). Professional resilience, defined as the ability to cope with difficulties, is of great importance, especially under stress and uncertainty that teachers frequently encounter (Djourova et al., 2020; Minten, 2020). Job satisfaction, on the other hand, refers to the satisfaction individuals get out of their jobs and has a strong link with transformational leadership due to its positive effect on creativity (Hidayat and Tjahjono, 2023; Tentama et al., 2020). Yildirim et al. (2024) revealed that professional resilience contributed to teachers' creativity. Psychological flexibility and professional resilience also help manage stress and uncertainty by increasing job performance and satisfaction. This study aims to fill the gap in the literature by investigating the mediating roles of professional resilience and job satisfaction to display how transformational leadership affects teachers' creative processes in the educational environment. The results of this study aim to help educational leaders better understand teachers' creative potential and develop leadership strategies that foster innovation and effectiveness (Al Harbi et al., 2019; Malik, 2024). Specifically, this research seeks to comprehensively explore the impact of transformational leadership on teachers' creativity. Investigating mediating variables such as professional resilience and job satisfaction provides deeper insights into how teachers can better cope with challenges and maintain their innovative capacities (Escortell et al., 2020; Ma et al., 2020). Building on existing research, the study systematically examines the relationships among these variables. Transformational leadership has been shown in prior studies to positively influence professional resilience and job satisfaction (Djourova et al., 2020; Tentama et al., 2020), which, in turn, serve as critical mediators for fostering creativity (Chen et al., 2021; Ripki et al., 2020). Based on this robust foundation in the literature, the hypotheses for the current study are formulated as follows:

*H1: Transformational leadership statistically significantly predicts teachers' professional resilience*.

*H2: Transformational leadership statistically significantly predicts teachers' job satisfaction*.

*H3: Transformational leadership statistically significantly predicts teachers' creativity*.

*H4: Teachers' professional resilience statistically significantly predicts creativity*.

*H5: Job satisfaction statistically significantly predicts teachers' creativity*.

*H6: Professional resilience mediates the association between transformational leadership and teachers' creativity*.

*H7: Job satisfaction mediates the association between transformational leadership and teachers' creativity*.

## Method

### Research design

Investigating the association between transformational leadership and teachers' creativity, the current study employed a correlational design. This research follows a cross-sectional survey design to collect data at a single point in time, providing a snapshot of the studied associations (Creswell, 2014). Mertens (2010) categorized correlational research into two groups: relational and prediction studies. Since the current study examined the performance of transformational leadership on teachers' creativity through the mediation of professional resilience and job satisfaction, it is a prediction study.

### Sample

The participants of this study included 417 teachers working in public and private schools in Istanbul. The study employed a simple random sampling method, in which each participant is equally likely to be selected, to increase the generalizability (Creswell, 2014). Of the participants, 240 were female (57.6%) and 177 were male (42.4%); 295 had an undergraduate degree (70.7%) and 122 had a graduate degree (29.3%). The experiences of the participating teachers were as follows: 106 teachers had an experience of 1–5 years (25.4%), 70 teachers 6–10 years (16.8%), 89 teachers 11–15 years (21.3%), 64 teachers 16–20 years (15.3%), and 88 teachers 21 years or over (21.1%). Of the teachers, 238 work in public schools (57.1%) and 179 in private schools (42.9%). As for the level they teach, 33 work in preschools (7.9%), 192 in primary schools (46.0%), 88 in secondary schools (21.1%) and 104 in high schools (24.9%). Lastly, 87 participants were aged between 20 and 30 years old (20.9%), 172 between 31 and 40 years old (41.2%), 101 between 41 and 50 years old (24.2%) and 57 between 51 and above (13.7%). The demographic diversity of the participants allows for a comprehensive examination of teachers' perceptions of creativity, leadership, satisfaction, and resilience (Büyüköztürk, 2010; Creswell and Poth, 2017).

### Data collection tools

The study employed four different data collection tools. The following section provides detailed information about these tools.

#### Transformational leadership scale

The scale was developed by Berger et al. (2012) and adapted into Turkish by Okan and Okan (2021). It is the short version with eight items loading on a single factor. The response options are as follows “(1) Strongly disagree”, “(2) Disagree”, “(3) Undecided”, “(4) Agree” and “(5) Strongly agree”. A sample item on the scale is “My administrator develops ways to motivate us”. Okan and Okan (2021) conducted exploratory and confirmatory factor analysis to reveal the scale's validity and Cronbach's Alpha internal consistency coefficient for reliability. The findings suggested that the scale had satisfactory psychometric properties.

In the current study, the researcher evaluated the validity and reliability of the scale. Confirmatory factor analysis was conducted for construct validity. The goodness-of-fit indices were as follows (Cmin/d*f* = 3.55; *p* = 0.00; CFI = 0.99; AGFI = 0.92; TLI = 0.98; RMSEA = 0.08; SRMR = 0.02), indicating that the uni-dimensional structure of the scale was validated (Hair et al., 2019). On the other hand, the factor loadings ranged between TL6 = 0.93-TL1 = 0.81 which were satisfactory. The Cronbach's Alpha internal consistency coefficient was α = 0.96 indicating a high level of reliability (Ho, 2006).

#### Professional resilience scale

The “Professional Resilience Scale” used in the study was developed by Näswall et al. (2019) to measure employees' ability to adapt to and manage changing conditions. Limon (2022) conducted the Turkish adaptation of the scale and modified the items for teachers. The uni-dimensional scale consists of 9 items which are responded on a 5-point Likert scale. On the scale, 1 correspond to “Almost never”, 2 “Rarely”, 3 “Sometimes”, 4 “Often” and 5 “Almost always”. A sample item is “I skillfully solve crisis at school”. Limon (2022) evaluated the validity of the scale through confirmatory factor analysis and reliability through Cronbach's Alpha internal consistency coefficient. The findings indicated that the scale had satisfactory psychometric properties.

The validity and reliability of the scale was also evaluated within the current study. Findings suggested that goodness of fit indices were (Cmin/d*f* = 3.98; *p* = 0.00; CFI = 0.96; AGFI = 0.91; TLI = 0.90; RMSEA = 0.09; SRMR = 0.04) indicating that the single factor structure of the scale was compatible with the current research data (Hair et al., 2019). On the other hand, the factor loadings ranged between TR7 = 0.57-TR9 = 0.78 which were satisfactory. Cronbach's Alpha internal consistency coefficient was α = 0.96, indicating high internal consistency (Ho, 2006).

#### Job satisfaction scale

The scale was developed by Brayfield and Rothe (1951) to measure individuals' satisfaction with their jobs. The version used in this study was the 5-item short form created by Judge et al. (1998). The scale was adapted into and validated in Turkish by Keser and Bilir (2019). The response options on the 5-point Likert type scale were as follows: (1) Strongly Disagree, (2) Disagree, (3) Neither Agree nor Disagree/No Opinion, Agree (4), and Strongly Agree (5). A sample item is “Every day at work feels like it will never end”.

The validity and reliability was also evaluated within this study. The goodness of fit indices were (Cmin/d*f* = 0.27; *p* = 0.85; CFI = 1.00; AGFI = 0.99; TLI = 1.00; RMSEA = 0.00; SRMR = 0.00) and the factor loadings ranged between SAT = 0.39 and SAT2 = 0.95 which suggested that the factor structure of the scale was consistent with the research data (Hair et al., 2019). The Cronbach's Alpha internal consistency coefficient was α = 0.82, indicating that the reliability criterion was satisfied (Ho, 2006).

#### Organizational creativity scale

To measure teachers' creativity, the researcher used individual creativity dimension of organizational creativity scale which was developed by Balay (2010). The scale consists of 16 items. The response options of the scale are “(1) Strongly Disagree”, (2) “Slightly Agree”, (3)“Moderately Agree”, “(4) Strongly Agree” and “(5) Totally Agree”. A sample item is “I try to go beyond existing limits to achieve new goals”.

The researcher conducted confirmatory factor analysis to evaluate the validity of the scale. The goodness of fit indices were (Cmin/d*f* = 4.30; *p* = 0.00; CFI = 0.92; AGFI = 0.85; TLI = 0.94; RMSEA = 0.09; SRMR = 0.04) and factor loadings ranged between IC1 = 0.60-IC12 = 0.82 showing that the single factor structure of the scale was compatible with the research data (Hair et al., 2019). The Cronbach's Alpha internal consistency coefficient was α = 0.94, indicating that the reliability criterion was satisfied (Ho, 2006).

### Data analysis

Data analysis was conducted on SPSS 25 and AMOS 24. Firstly, the data set was scanned for missing data and no missing data was found. Secondly, the distribution of the data was analyzed through kurtosis-skewness coefficients. The findings presented in Table 1 showed that the data had a normal distribution (Mertler and Vannatta, 2017). Drawing on these findings, parametric tests were employed in data analysis. The research model was tested through Structural equation modeling. Thus, the steps suggested by Hair et al. (2019) were followed. First, the measurement model was designed and its validity was evaluated. Then, the validity of the structural model was evaluated. Since transformational leadership, teacher professional resilience and job satisfaction are predictors of creativity in the model, whether there was a multicollinearity problem among these three variables was investigated calculating VIF, Tolerance and Condition Index. The findings emerged as (Tolerance = 0.83; VIF = 1.20; Condition Index=10 for transformational leadership), (Tolerance = 0.88; VIF = 1.14; Condition Index = 12.23 for teacher professional resilience), and (Tolerance = 0.86; VIF = 1.17; Condition Index = 19.40 for job satisfaction). These findings demonstrated that there was no multicollinearity problem among the predictor variables (Mertler and Vannatta, 2017). On the other hand, multivariate kurtosis and CR values were calculated to test the assumption of multivariate normality and they emerged as Multivariate kurtosis = 225.44 and CR = 41.75. Since these findings indicated that the assumption of multivariate normality was not satisfied, the model was tested using the bootstrap technique with 5,000 resamples and 95% confidence interval (Collier, 2020).

**Table 1:** Descriptive statistics and correlations.

**Descriptive**						**Correlations**			
**Variable**	***N***	**Mean**	**SD**	**Skewness**	**Kurtosis**	**(1)**	**(2)**	**(3)**	**(4)**
(1) Leadership	417	3.64	0.94	−0.80	0.34	–	0.34^**^	0.30^**^	0.26^**^
(2) Satisfaction	417	3.72	0.77	−0.53	−0.27		–	0.26^**^	0.25^**^
(3) Resilience	417	3.96	0.58	−0.18	−0.10			–	0.73^**^
(4) Creativity	417	3.87	0.63	−0.29	0.17				–

** p is significant at 0.00 level.

### Ethics

Data collection permission was obtained from Haliç University Ethics Committee with the letter dated 12.06.2024 and numbered 05. The data collection tool was distributed online *via* school administration channels over a 2 month period (June–August 2024) to ensure accessibility and broad participation. The data was collected complying with local legislation and institutional requirements. Participants gave their written informed consent to respond to the scales.

## Findings

The study aim to test the hypothesized relationships between transformational leadership, professional resilience, job satisfaction, and creativity among teachers. Structural equation modeling (SEM) was used to examine both direct and mediating effects. Descriptive statistics, correlations, and hypothesis testing results are presented below to provide a comprehensive overview of the study's findings.

Table 1 presents the descriptive findings for the variables and their associations. The results show that participants reported the following mean scores: transformational leadership (*M* = 3.64, SD = 0.94), job satisfaction (*M* = 3.72, SD = 0.77), professional resilience (*M* = 3.96, SD = 0.58), and creativity (*M* = 3.87, SD = 0.63). These descriptive statistics are based on aggregated responses obtained from standardized instruments, ensuring objectivity in measuring participants' perceptions (Hair et al., 2019). The findings indicate that teachers generally held positive perceptions of the research variables. In terms of associations, transformational leadership exhibited a significant positive correlation with job satisfaction (*r* = 0.34, *p* < 0.01), professional resilience (*r* = 0.30, *p* < 0.01), and creativity (*r* = 0.26, *p* < 0.01). Furthermore, job satisfaction was significantly associated with both professional resilience (*r* = 0.26, *p* < 0.01) and creativity (*r* = 0.25, *p* < 0.01). Notably, professional resilience demonstrated a strong positive correlation with creativity (*r* = 0.73, *p* < 0.01). In conclusion, these findings indicate the presence of significant, positive, medium and high-level associations between variables.

### Findings on the measurement model

First the measurement model, which included covariates among all latent variables, was evaluated. The results indicated that the goodness-of-fit indices were as follows (Cmin/d*f* = 3.05, *p* < 0.01; CFI = 0.88; AGFI = 0.78; SRMR = 0.06; TLI = 0.87; RMSEA = 0.07). Additionally, the factor loadings for the observed variables ranged from TL6 = 0.92 to SAT3 = 0.45, with all significance levels being *p* < 0.00. These findings suggested that the measurement model had adequate fit.

### Findings on structural equation modeling

**Figure 1:**
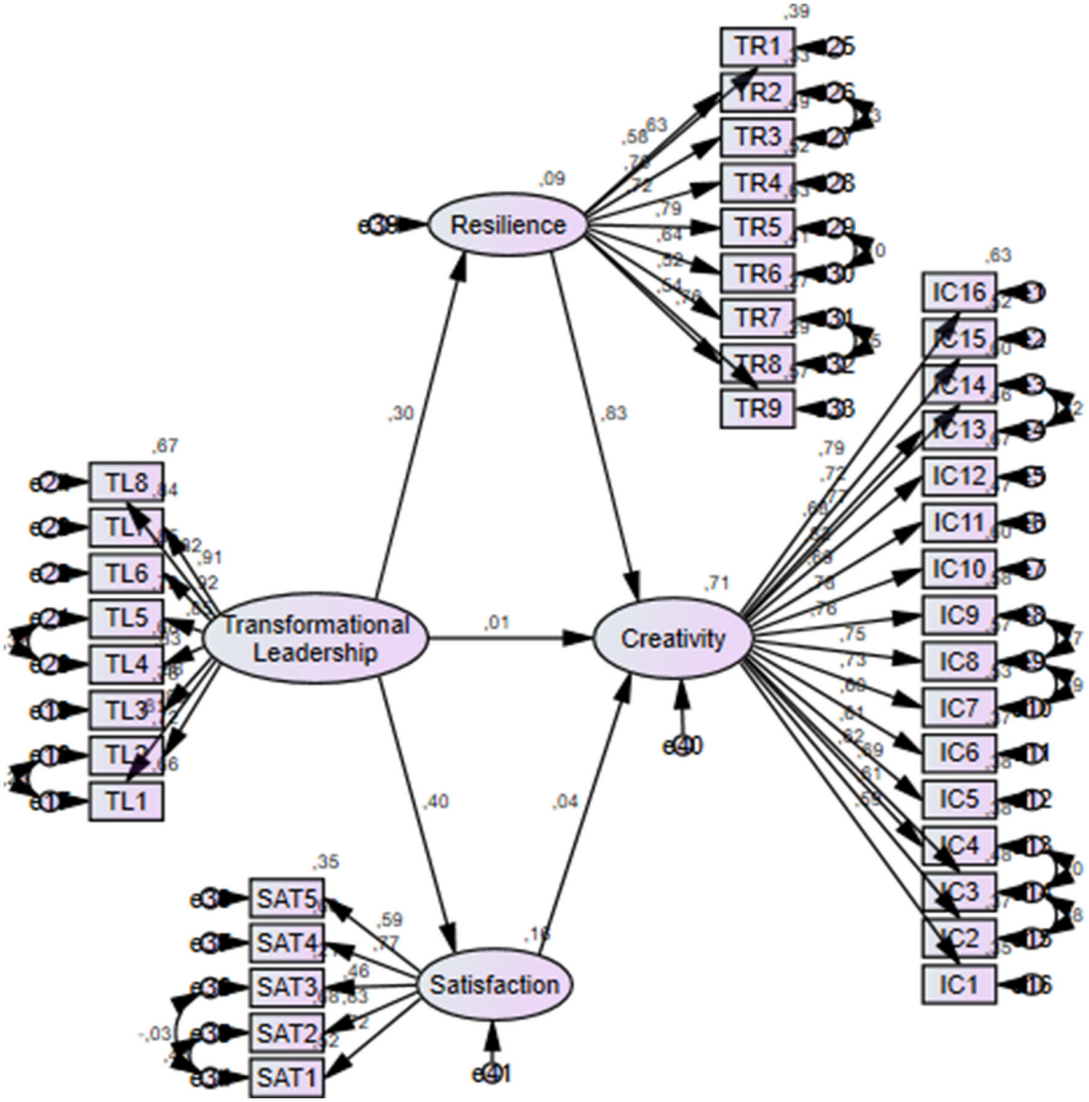
Structural equation model.

Table 2 demonstrates the standardized direct and indirect effects. The findings showed that transformational leadership statistically significantly predicted teachers' professional resilience *(*β = 0.30; *p* < 0.01) and job satisfaction *(*β = 0.41; *p* < 0.01). However, the direct effect of transformational leadership on creativity was not statistically significant *(*β = 0.01; *p* = 0.74). Additionally, teachers' professional resilience significantly predicted creativity *(*β = 0.47; *p* < 0.01), while the effect of job satisfaction on creativity was not statistically significant *(*β = 0.04*; p* = 0.47). Furthermore, the effect of transformational leadership on creativity through professional resilience was statistically significant (β = 0.18; *p* < 0.01), whereas the effect through job satisfaction was not statistically significant *(*β = 0.01; *p* = 0.44). Figure 1 illustrates the model of the study.

**Table 2:** Standardized direct and indirect effects.

**Bootstrap 5,000 (95% CI)**						
**Paths**	β	**SE**	**LB**	**UB**	***p***	
**Direct Paths**						***Hypothesis***
(1) TL → TR	0.30	0.05	0.29	0.52	*0.00*	Confirmed
(2) TL → JS	0.41	0.06	0.19	0.40	*0.00*	Confirmed
(3) TL → C	0.01	0.04	−0.07	0.09	0.74	Unconfirmed
(4) TR → C	0.83	0.03	0.78	0.88	*0.00*	Confirmed
(5) JS → C	0.04	0.05	−0.06	0.12	0.47	Unconfirmed
**Indirect Effects**						
(6) TL → TR → C	0.18	0.04	0.11	0.26	*0.00*	Confirmed
(7) TL → JS → C	0.01	0.01	−0.02	0.04	0.44	Unconfirmed

*p* is significant at 0.01 level.

## Discussion

This study primarily examined whether professional resilience and job satisfaction mediated the relationship between transformational leadership and creativity. While transformational leadership significantly influenced professional resilience and job satisfaction, its direct impact on creativity was not observed. This finding aligns with Amabile (2018), who suggests that creativity is often driven by intrinsic motivation. Similarly, the results support previous studies emphasizing the critical role of leadership in fostering a supportive environment conducive to creativity and resilience (Leithwood and Sun, 2012; Djourova et al., 2020). Practical implications include the importance of leadership training programs that develop transformational skills, which can enhance teachers' creative potential and job satisfaction (Rahmatika and Saragih, 2023; Ripki et al., 2020). Additionally, as Kandemir (2024) and Jiatong et al. (2022) emphasize, transformational leadership significantly reduces workplace exclusion by fostering effective school environments. This reduction in exclusionary practices strengthens collaboration among teachers, indirectly promoting creative and innovative pedagogical practices. Furthermore, the study investigated teachers' perceptions of the variables. The findings indicated that teachers' perceptions of transformational leadership were generally positive, suggesting that school principals adopted this leadership style effectively. Bass and Avolio (1994) stated that transformational leadership was a factor that positively contributed to organizational outcomes by increasing both employees' motivation and commitment. On the other hand, the findings showed that teachers were satisfied with their jobs. Judge and Piccolo (2004) found that transformational leaders enhanced employees' job performance and commitment through job satisfaction. The findings in this study also suggested that the relationship between transformational leadership and job satisfaction was significant and positive which was consistent with the previous research. As for the teachers' professional resilience and creativity, the findings showed that teachers perceived themselves resilient and creative. On the other hand, these two variables were strongly and positively associated. Previous research is consistent with this finding which showed that higher professional resilience resulted in higher creativity. Carmeli and Schaubroeck (2007) stated that resilience increased individuals' capacity to cope with stress and this contributed positively to creativity. Shin and Zhou (2003) emphasized that supportive leadership styles enhanced creative processes. Recent studies emphasize that transformational leadership fosters innovative practices by enhancing teachers' professional resilience (Rahmatika and Saragih, 2023; Fareed et al., 2022). This aligns with findings that highlight leadership's indirect role in promoting creativity through mediating factors like resilience and job satisfaction (Jiatong et al., 2022; Hidayat and Tjahjono, 2023).

The first hypothesis in this study suggested that transformational leadership positively affected teachers' professional resilience and the findings confirmed the hypothesis. This suggests that leaders who inspire, motivate, and provide individualized support are more likely to enhance teachers' resilience, enabling them to adapt to challenges effectively. This result aligns with prior research by Djourova et al. (2020), which highlights the positive impact of transformational leadership on employee adaptability. There is abundant empirical evidence in the literature that transformational leaders increased employees' resilience and capacity to cope with stress (Luthans et al., 2007; Zacher and Johnson, 2015). For example, Avey et al. (2010) found that transformational leadership strengthened employees' performance by increasing their psychological capital and resilience. Additionally, Walumbwa et al. (2008) stated that transformational leadership had a positive influence on stress management and resilience. The second hypothesis was that transformational leadership positively predicted teachers' job satisfaction. The findings showed that transformational leadership had a significant influence on job satisfaction. This finding is consistent with the existing literature (Kovjanic et al., 2013; Lai et al., 2020). Judge and Piccolo (2004) demonstrated that transformational leadership positively contributed to job satisfaction. On the other hand, Dilekçi (2022) revealed that teacher autonomy had a positive influence on job satisfaction and creative processes which implied that teachers with higher autonomy were more creative, self-confident and motivated. In another study, Chan (2019) found that transformational leaders strengthened employee engagement and job satisfaction. The third hypothesis of the study suggested that transformational leadership predicted teachers' creativity. However, the findings showed that transformational leadership did not have a significant effect on teachers' creativity. This finding is consistent with the previous research arguing that transformational leadership indirectly affected creativity. For example, Shin and Zhou (2003) stated that although transformational leaders provided a supportive environment for creative processes, they did not have a direct influence on creativity. Amabile (2018) argued that creativity was more associated with intrinsic motivation. In this sense, the findings of the current study coincide with existing literature (Gong et al., 2009; Kark and Shamir, 2013; Zhou and George, 2001). The fourth hypothesis was that professional resilience predicted teachers' creativity. The findings showed that professional resilience had a significant influence on creativity which was consistent with the existing literature (Gong et al., 2009; Luthans et al., 2007). For example, Carmeli and Schaubroeck (2007) revealed that resilient individuals were more successful in creative processes. In another study, Rego et al. (2012) showed that resilient individuals had a higher capacity to raise innovative and creative solutions. The fifth hypothesis was that job satisfaction predicted teachers' creativity which was not confirmed by the findings. The previous literature suggested that job satisfaction had a limited influence on creativity. Amabile (2018) suggested that creativity was more associated with intrinsic motivation and job satisfaction did not have a direct effect on creativity. Similarly, Zhou and George (2001) stated that job satisfaction enhanced creativity indirectly (Tierney and Farmer, 2002; Carmeli and Schaubroeck, 2007; Gong et al., 2009). The sixth hypothesis of the study was that professional resilience mediated the relationship between transformational leadership and teachers' creativity which was confirmed by the findings. This finding is also consistent with the previous literature. Luthans et al. (2007) indicated that transformational leaders contributed to creative processes by increasing employees' resilience. There are also studies showing that transformational leaders support creativity through professional resilience (Walumbwa et al., 2008; Zacher and Johnson, 2015). These results are consistent with recent studies, which highlight the mediating role of professional resilience in enhancing creativity within educational settings (Chen et al., 2021; Ripki et al., 2020). The last hypothesis suggested that job satisfaction mediated the relationship between transformational leadership and teachers' creativity which was not confirmed by the findings. The existing literature also suggested that the influence of job satisfaction on creativity was limited (Alzoraiki et al., 2024; Zhang and Bartol, 2010). These findings are consistent with this study.

## Conclusion

This study investigated the associations between transformational leadership, job satisfaction, professional resilience and creativity, and particularly revealed a strong positive relationship between professional resilience and creativity. Findings showed that transformational leadership positively influenced both job satisfaction and professional resilience. Professional resilience had a strong effect on creativity and transformational leadership was indirectly effective in this process. However, the direct effect and mediating role of job satisfaction on individual creativity remained limited. These results show that transformational leadership practices in educational organizations can support the teachers' creativity boosting their professional resilience and emphasize that this leadership style plays a critical role in increasing teachers' performance and satisfaction.

## Limitations

The generalizability of these findings is limited to the city where the data was collected and teachers as the participants of the study. Secondly, the data collection tools were self-reported measures which might lead to social desirability bias. Thirdly, since the research had a cross-sectional design, it does not support the conclusions on causal relationships. Despite these limitations, research findings provide significant insights into the relationships between transformational leadership, job satisfaction, professional resilience and creativity in educational organizations.

## Recommendations

The findings of this study have a number of important implications for future practice. They provide significant insights into how leadership practices and professional development of teachers can be supported. Adopting a transformational leadership style can positively contribute to teachers' job satisfaction and creativity by increasing their professional resilience. School administrators can enhance their transformational leadership skills through training, thereby creating a working environment that strengthens teachers' job performance, commitment, and innovation abilities. Specific strategies include providing regular feedback to teachers, fostering a collaborative school culture, and offering professional development programs focused on stress management and innovative teaching methods. Additionally, creating flexible working conditions and promoting leadership practices that prioritize individual teachers' needs can further improve educational outcomes. Considering the positive effect of professional resilience on teachers' creativity, professional development programs that strengthen teachers' ability to cope with stress can be organized which can support teachers' creative thinking capacities by increasing their ability to cope with challenges. Finally, schools should allow for more practices that enhance job satisfaction and professional resilience. Giving regular feedback to teachers, creating a supportive school culture and flexible working conditions that will encourage teachers' creativity will increase the overall quality of education and strengthen teachers' commitment.

## Data Availability

The original contributions presented in the study are included in the article/supplementary material, further inquiries can be directed to the corresponding author.
